# Dysregulation of Iron Metabolism in Alzheimer's Disease, Parkinson's Disease, and Amyotrophic Lateral Sclerosis

**DOI:** 10.1155/2011/378278

**Published:** 2011-10-12

**Authors:** Satoru Oshiro, Masaki S. Morioka, Masataka Kikuchi

**Affiliations:** ^1^Division of Cell Biology, Department of Health Science, Graduate School of Sports and Health Science, Daito Bunka University, 560 Iwadono, Higashi-Matsuyama, Saitama 355-8501, Japan; ^2^Department of Cardiovascular Medicine, The University of Tokyo Graduate School of Medicine, 7-3-1 Hongo, Bunkyo-ku, Tokyo 113-8655, Japan; ^3^Department of Bioinformatics, Graduate School of Medical and Dental Sciences, Tokyo Medical and Dental University, Yushima, Bunkyo-ku, Tokyo 113-8510, Japan

## Abstract

Dysregulation of iron metabolism has been observed in patients with neurodegenerative diseases (NDs). Utilization of several importers and exporters for iron transport in brain cells helps maintain iron homeostasis. Dysregulation of iron homeostasis leads to the production of neurotoxic substances and reactive oxygen species, resulting in iron-induced oxidative stress. In Alzheimer's disease (AD) and Parkinson's disease (PD), circumstantial evidence has shown that dysregulation of brain iron homeostasis leads to abnormal iron accumulation. Several genetic studies have revealed mutations in genes associated with increased iron uptake, increased oxidative stress, and an altered inflammatory response in amyotrophic lateral sclerosis (ALS). Here, we review the recent findings on brain iron metabolism in common NDs, such as AD, PD, and ALS. We also summarize the conventional and novel types of iron chelators, which can successfully decrease excess iron accumulation in brain lesions. For example, iron-chelating drugs have neuroprotective effects, preventing neural apoptosis, and activate cellular protective pathways against oxidative stress. Glial cells also protect neurons by secreting antioxidants and antiapoptotic substances. These new findings of experimental and clinical studies may provide a scientific foundation for advances in drug development for NDs.

## 1. Introduction

Iron (Fe) is an essential but a possibly hazardous micronutrient for animals, plants, humans, and microorganisms. Mammalian cells require iron for fundamental biochemical activities such as oxygen transport, energy metabolism, and DNA synthesis. In humans, iron stores are mainly distributed among reticulocytes, macrophages, and the liver [1], and the balance between iron absorption and output is controlled by the intestine [2].

 Dietary nonheme iron is generally found in the ferric (Fe^3+^) state, and it requires to be reduced to the ferrous (Fe^2+^) state for absorption. This reduction from Fe^3+^ to Fe^2+^ is mediated by a ferric reductase [3, 4] called duodenal cytochrome b [5, 6], which resides on the luminal surface of intestinal absorptive cells. Once reduced, Fe^2+^ is transported across the apical membrane of enterocytes by the divalent metal transporter 1 (DMT1) [7]. Internalized Fe^2+^ is processed by enterocytes and eventually exported across the basolateral membrane into the bloodstream via the solute carrier and Fe^2+^ transporter, ferroportin 1 (FPN1) [8]. Fe^2+^ is then oxidized to Fe^3+^ via the membrane-bound ferroxidase, hephaestin, which physically interacts with FPN, and its plasma homologue, ceruloplasmin (CP) [9]. The FPN1-mediated efflux of Fe^2+^ from enterocytes into the plasma is critical for systemic iron homeostasis. This process is negatively regulated by hepcidin, a liver-derived peptide hormone that binds to FPN1 and promotes its phosphorylation, internalization, and lysosomal degradation [10]. Iron is delivered to tissues by circulating transferrin (Tf), a transporter that captures iron released in plasma mainly from intestinal enterocytes or reticuloendothelial macrophages. Specifically, dietary iron excreted from enterocytes and reticulocytes binds to Tf in a process catalyzed by CP and is delivered to all organs and tissues, including the brain cells. Circulating iron-laden Tf binds with high affinity to the Tf receptor 1 (TfRl) on the cell surface, resulting in endocytosis and internalization of the TfR-Tf-iron complex in the cell. A proton pump promotes acidification of the endosome, triggering the release of Fe^3+^ from Tf that remains bound to TfR1. Fe^3+^ is reduced to Fe^2+^, which is transported across the endosomal membrane by DMT1 to the cytosol where it can be sequestered by ferritin. Iron can also be directly transported to the mitochondria where it is utilized for energy synthesis or formation of iron sulfur clusters for iron sensing. In cells, the best-characterized iron-sensing mechanism is the result of posttranscriptional regulation of mRNA. Sequences of mRNA called iron-responsive elements (IREs) are contained within mRNA sequences that encode ferritin and TfR. IRE-binding proteins (IRE-BP1 or IRE-BP2) bind to these mRNA sequences and regulate post-transcriptional expression of these iron metabolism-related mRNAs. Specifically, when cellular iron levels are low, IRE-BP exists as a 3Fe-4S iron sulfur cluster and binds to IREs located in the 5′ and 3′ untranslated regions (UTRs) of ferritin and TfR mRNA, respectively. This binding inhibits translation of the iron storage protein, ferritin. When cellular iron levels are low, IRE-BP containing the 3Fe-4S cluster also stabilizes TfR mRNA, causing an increase in the mRNA translation of TfR proteins. Once generated, these proteins are embedded in the cell's plasma membrane to promote cellular iron uptake. When cellular iron levels are high, IRE-BP changes to the alternate 4Fe-4S cluster; in this form, its mRNA-binding activity is repressed, and IRE-BP is released from IRE. Without the binding of IRE-BP (4Fe-4S), ferritin mRNA undergoes translation. TfR mRNA is rapidly degraded, decreasing translation, which results in the production of fewer TfR proteins. Other known genes that encode IREs in their mRNA 5′-UTRs include DMT1, 5-aminolevulinic acid synthetase, hypoxia-inducible factor 2a (HIF-2a), and amyloid precursor protein (APP). Recent studies have shown that IRE-BP2 is degraded by SKP1-CUL1-FBXL5 ubiquitin ligase protein complex in HEK293 cells [11, 12]. This complex directly senses iron level through FBXL5, a member of the F-box family of adaptor proteins that has iron-binding hemerythrin-like domains at its N terminus. Thus, iron homeostasis also involves a proteolytic pathway that couples with IRE-BP2-dependent translational regulation in cells.

## 2. Iron Metabolism in the Brain

**Figure 1:**
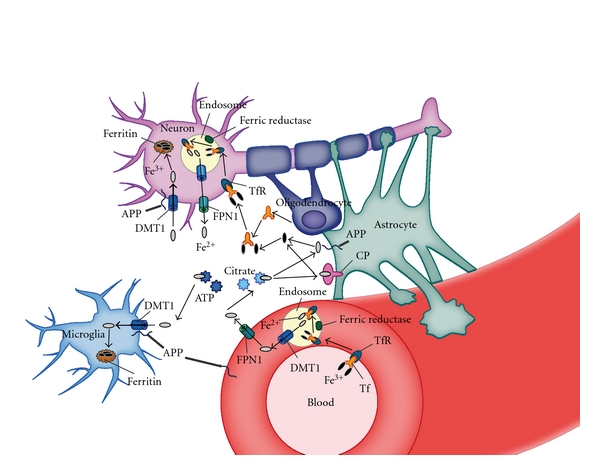
Iron metabolism in the brain. Transferrin (Tf) bound to ferric iron (Fe^3+^) is taken up by the Tf receptor (TfR) in the luminal membrane of endothelial cells, and Fe^3+^-laden Tf and TfR complex are internalized into endosomes. Fe^3+^-bound Tf is reduced by duodenal cytochrome b to ferrous iron (Fe^2+^). Fe^2+^ may be transported to the cytosol by the divalent metal transporter l (DMT1) in the endosomal membrane and exported into the extracellular fluid by ferroportin l (FPN1). FPN1 interacts with the amyloid precursor protein (APP), which may function as a ferroxidase at the plasma membrane of neurons, microglia, and astrocytes. After Fe^2+^ release, Tf is recycled to bind to Fe^3+^ in the blood. Ceruloplasmin (CP) on the membrane of astrocytes oxidizes Fe^2+^ to Fe^3+^ to bind for subsequent binding to Tf. Neurons take up Tf-bound and non-Tf-bound iron (NTBI). NTBI may also bind to citrate and ATP derived from astrocytes to function as a source of iron for oligodendrocytes and astrocytes. Oligodendrocytes synthesize Tf, which play a role in intracellular transport. The cytosolic iron storage protein ferritin traps and stores NTBI in the brain cells.

Iron uptake in the brain is mediated by the expression of TfR on the luminal endothelial surface in the blood-brain barrier. As shown in Figure 1, TfR binds to Tf-bound Fe^3+^ derived from diet and excreted from enterocytes and reticulocytes. This is followed by internalization of the Tf-TfR complex into endosomes. Acidification of the endosome facilitates the release of Fe^3+^, which is reduced by a ferric reductase to Fe^2+^ [1, 3, 13]. Once Fe^2+^ is transported into the cytosol of endothelial cells by endosomal DMT1 [13], it is exported into CNS by FPN1. After Fe^2+^ release, TfR is recycled to bind iron-laden Tf in the blood.

Neurons express TfR, DMT1, APP, FPN1, and ferritin as iron regulators but do not express CP. Therefore, neurons take up both Tf-bound and non-Tf-bound iron (NTBI). Interestingly, a novel iron-regulatory signaling cascade in neurons called the N-methyl D-aspartate (NMDA) receptor-nitric oxide (NO) signaling pathway physiologically induces iron uptake via glutamine-NMDA activation [14]. Downstream of this cascade, DMT1 physically interacts with peripheral benzodiazepine receptor-associated protein 7, which plays a role in signaling with S-nitrosylation of Dexras1 from the NMDA receptor at both the endosome and plasma membrane. Moreover, Cheah et al. showed that overactivation of NMDA receptors leads to neurotoxicity, which is prevented by iron chelation.

Astrocytes specifically express CP [15] and oxidize Fe^2+^ to Fe^3+^ following binding to Tf. Although astrocytes are the primary cells in CNS that express Tf, oligodendrocytes also express Tf [16]. In cases where Tf is highly saturated by iron, NTB1 may be present in significant amounts and may function as an important iron source for cells such as oligodendrocytes, which do not express much TfR1. NTBI binds to citrate [17] or ATP derived from astrocytes to function as a source of iron for oligodendrocytes and astrocytes. Microglia express DMT1, APP, and ferritin, suggesting that glial cells assist neurons in maintaining iron homeostasis in the brain environment. Once abnormal iron homeostasis induces oxidative stress in the brain, neurons are exclusively damaged by the resulting neurotoxicity, leading to the development of neurodegenerative diseases (NDs). However, the glial cells with diverse functions may regulate iron metabolism to protect the normal function of neurons. Elucidation of the mechanisms underlying the interaction between neurons and glial cells in the brain may lead to a better understanding of iron homeostasis within the cell, and its dysregulation in NDs.

## 3. Iron Metabolism and Neurodegenerative Diseases

### 3.1. Iron Metabolism in Alzheimer's Disease

Alzheimer's disease (AD) is a slowly progressing disease of the brain characterized by impairment of memory and disturbances in reasoning, planning, language, and perception. The main risk factor for AD is advanced age. Classic pathological features of AD are aggregates of insoluble amyloid beta-protein (Ab), and neurofibrillary tangles (NFTs) consisting of precipitates or aggregates of hyperphosphorylated tau protein. Fe^3+^ binds to hyperphosphorylated tau protein and is reduced to Fe^2+^, which induces NFT production [18]. Redox-active iron (Fe^2+^/Fe^3+^ complexes) is deposited near the Ab plaques and NFT in the cortex of AD brains [19]. Ab triggers neuritic degradation in both neurites and synapses [20, 21]. APP, a type I transmembrane protein and a well-known precursor protein of Ab, is ubiquitously localized in neuron, glial, and the other cells [22, 23] (Figure 1).

The presence of IRE in APP mRNA suggests an association between iron metabolism and AD [24]. APP IRE is homologous with the canonical IRE RNA stem loop that binds IRE-BP1/IRE-BP2 in order to regulate intracellular iron homoeostasis by modulating ferritin mRNA translation and TfR mRNA stability. APP IRE interacts with IRE-BP1, whereas the heavy subunit of ferritin (H-ferritin) IRE binds to IRE-BP2 in neural cell lines, human brain cortex tissue, and human blood lysates [25]. Both IRE-BPs are implicated in iron regulation in the brain; IRE-BP1 binds to APP IRE, [26] and IRE-BP2 upregulates APP gene expression in the hippocampus of IRE-BP2 mice (−/−) [27]. Recently, Duce et al. showed that a motif within the E2 domain of APP that is homologous to H-ferritin possesses ferroxidase activity. In addition, APP interacts with FPN and assists in iron efflux to extracellular fluid [23]. Moreover, alpha-cleavage of APP is regulated by iron loading *in vitro*, suggesting that iron-mediated oxidative stress causes APP misregulation [28]. However, it is unclear if APP misregulation is the underlying cause of AD and other NDs. Furthermore, APP and CP mRNAs are translationally regulated by proinflammatory cytokines: interleukin-1 (IL-1) and interferon gamma, respectively [29, 30], indicating an association between AD and inflammatory responses within the brain that are primarily performed by activated microglia. Thus, examining the effect of iron on the interactions between neurons and microglial cells may provide us with the physiological causes underlying AD pathogenesis.

### 3.2. Iron Metabolism in Parkinson's Disease

Parkinson's disease (PD) is an ND caused by dopaminergic neuronal cell death in the substantia nigra (SN). The main symptoms of PD patients are tremors in the hands and feet, bradykinesia, rigidity, and postural instability. The neuropathological features of PD are intracytoplasmic inclusions in SN, known as Lewy bodies, which are widely distributed throughout paralimbic and neocortical regions. Phosphorylated a-synuclein is the main component of Lewy bodies. A decrease in the number of neurons in SN leads to an increase in acetylcholine levels in the striatum, resulting in the collapse of normal homeostasis. Iron deposition has been found in dopaminergic neurons within SN [31–33]. SN also synthesizes neuromelanin, which is an insoluble pigment derived from cytosolic catechols present in the granule form. Neuromelanin is associated with iron storage and binds iron-forming stable complexes to sequester large amounts of iron in dopaminergic neurons, resulting in elevated iron levels. According to progression of aging as a risk factor, melanin pigments decrease in SN. Thus, iron chelation and sequestration are effective to protect neurons. Excess iron in dopaminergic neurons can accelerate toxic a-synuclein fibril formation, leading to cellular dysfunction [34]. Ferritin and neuromelanin may contribute to neuroprotection [35], since free cytosolic iron can trigger oxidative stress and promote a-synuclein deposition in Lewy bodies [24, 36]. Iron chelation and overexpression of iron-sequestering ferritin have protective roles in animal PD models [24].

A recent *in vitro* study reported that upregulation of DMT1 without IRE induced by L-3, 4-dihydroxyphenylalanine (L-DOPA), and the resulting increase in DMT1-IRE-mediated iron influx play a key role in L-DOPA-induced neurotoxicity in cortical neurons [37]. More importantly, elevated expression of a DMT1 isoform has been found in SN of PD patients [38], which may be associated with iron accumulation. Similarly, in 1-methyl-4-phenyl-1,2,3,6-tetrahydropyridine-treated mice, a PD model, increased DMT1 expression was found in the ventral mesencephalon, followed by corresponding iron deposition and dopaminergic cell loss. Thus, DMT1 plays an instrumental role in iron accumulation and subsequent oxidative stress-mediated cell damage. Neurons and glial cells appear to export iron via a GPI-anchored form of CP. Quite recently, Jin et al. reported that a decrease in serum CP levels may specifically promote iron deposition in SN of PD patients [39]. Genetic and pharmacological experiments have proven that chelation of excess iron may be an effective therapy for PD [40]. These findings have led to the development of therapeutic iron chelators for the treatment of NDs (see Section 4. “Iron Chelation Therapy for Neurodegenerative Diseases”).

### 3.3. Iron Metabolism in Amyotrophic Lateral Sclerosis

Similar to AD and PD, excess iron levels have also been observed in the central nervous system (CNS) of amyotrophic lateral sclerosis (ALS) patients [41]. Abnormal iron levels can cause inflammation, microglia activation, and oxidative damage in the brain tissue, and this is further supported by reports that show altered expression of several iron regulators (i.e., TfR1, DMT1, FPN1, H-ferritin, mitochondrial ferritin, and IRE-BP1) in ALS [42]. However, abnormalities of other iron factors such as TfR2, NM, CP, and Tau have never been reported in ALS, indicating that the ALS pathophysiology involves mechanisms of iron dysregulation different from those of other NDs. One of the most frequently reported gene mutations in ALS involves superoxide dismutase 1 (SOD1), a free radical scavenger, which was identified in about 20% of familial ALS cases, for an overall ALS incidence of about 2%. From our original searching results, 127 different mutations of SOD1 gene were identified in a total of 172 cases in the ALS database [43]. The SOD1 enzyme catalyzes the conversion of O_2_^−^ to O_2_ and H_2_O_2_, and consequently dysfunctional SOD1 results in imbalance between free radicals and ions, ultimately culminating in increased cellular oxidative stress. The potential metabolic consequences of this are wide ranging, affecting the cellular processes such as mitochondrial electron transport, cell proliferation, and iron metabolism. Recent reports showed that the human ataxia type 2 (ATXN2) gene, a polyglutamine disease gene mutated in spinocerebellar ataxia type 2 (SCA2), was associated with an increased risk for ALS [44]. In 915 cases that were examined, 4.7% of ALS patients showed expansion of CAG repeat sequences in the ATXN2 gene. ATXN2 directly interacts with the 43-kDa transactive-response- (TAR-) DNA-binding protein (TDP-43), a protein that binds to the TAR-DNA element of human immunodeficiency virus type 1 and regulates RNA splicing [45]. Pathologically, TDP-43 is also found as cytosomal-ubiquitinated inclusions in frontotemporal lobar dementia (FTLD), some familial ALS, and a few sporadic cases. When cocultured neural and spinal cord cells are exposed to agents inducing cellular stresses such as oxidative stress (H_2_O_2_), ER stress (thapsigargin), or proteasome inhibition (epoxomicin), TDP-43 translocates from the nucleus to the cytosol. This is accompanied by the inactivation of cell survival signaling pathways that involve mitogen-activated protein kinase (MAPK)/extracellular signal-regulated kinases (ERK1/2) [46]. Together, these results suggest that cellular stressors are the key factors associated with the dysfunction of ATXN2 and TDP-43 in the pathogenesis of ALS. According to the ALS mutation database (https://reseq.lifesciencedb.jp), there are additional ALS causative genes, including vesicle-associated membrane protein-associated protein B and C (VAMP), fusion (FUS), dynactin 1 (DCTN1), and ALS2 genes and are a total 27 ALS-related genes. While the functional relationship between these genes and ALS is still unclear, the results are consistent with a recently conducted genome-wide analysis of the gene expression profiles of glial cells and motor neurons in two common ALS mouse models [47]. This study revealed that in the most significant gene sets, the functions of ALS-related genes are commonly associated with protein modification/phosphorylation, muscle contraction regulation, and stress responses or are involved in glial cell-related functions (immune response).

The current experimental evidences in ALS studies indicate the importance of both response to oxidative stress and inflammatory systems for pathophysiological mechanism of NDs [17, 48, 49]. Interestingly, recent studies have demonstrated that the macrophage-recruiting chemokine, monocyte chemoattractant protein 1 (MCP-1 or CCL2), is upregulated in the glial cells of spinal cord tissues in SOD1^G93A^ transgenic ALS mice [48], implicating the role of the brain's innate immune system and related inflammation in ALS pathophysiology. As one of the effector cells of brain innate immune system, microglia regulate both oxidative stress and inflammatory responses to cytokines and chemokines within the nervous system and regulate iron homeostasis [49]. Consistent with this, we demonstrated that a moderate neural protective effect against excess NTBI is seen when neurons and microglia are cocultured without astrocytes [17], suggesting that in ALS the interaction between neurons and microglia is fundamentally related to responses to oxidative stress and inflammation.

## 4. Iron Chelation Therapy for Neurodegenerative Diseases

As accumulating evidence indicates the role of iron dysregulation in causing neuronal cell death in the pathophysiology of NDs, it follows that iron chelators may be promising agents for clinical treatment of NDs. Treatment with iron chelators aims at removing excess iron that is the cause of neurotoxicity in brain tissue, and currently several oral, subcutaneous, and intraperitoneal iron-chelator drug formulations are already being utilized in the clinical treatment of NDs [50].

Desferrioxamine (DFO), a natural iron scavenger isolated from *Streptomyces pilosus, *was developed in 1961 for the treatment of hemochromatosis and was the first-designed iron chelation drug to be manufactured [51]. In AD and PD, DFO chelates both aluminum and iron ions and protects against 6-hydroxydopamine-induced damage of dopaminergic neurons [52, 53]. However, treatment with DFO requires continuous dosing due to its short circulating half-life. Furthermore, the efficiency of DFO in PD has been also limited because of its inability to penetrate the blood-brain barrier [54]. Side effects of DFO administration include injection site reactions and retinal toxicity as reported by Bring et al. [55]. Consequently, treatment with DFO has been regarded to negatively affect the quality of life (QOL) of ND patients. 

Deferiprone (DFP or L1) is an oral Fe^2+^-specific chelating agent used to treat iron accumulation in thalassaemia. Clinical trials of treatment with L1 for Friedreich's ataxia (FA), a severe inherited neurological disease, demonstrated the elimination of labile iron deposits in a specific brain region without significant adverse changes in hemoglobin or plasma iron levels [56]. An additional advantage of L1 is its cost effectiveness, compared to DFO as it does not require the same long-term continuous dosing [57]. Side effects of treatment with L1 include agranulocytosis, hepatotoxicity, zinc deficiency [58], and possible Ll-induced overchelation of iron in cells despite normal levels. 

The efficacy and safety of several combinations of iron chelation therapies has also been examined in recent clinical studies, which aimed to primarily improve the QOL of patients. Indeed, previous reports showed that combination of DFO and L1 therapy was more effective and less burdensome for the *β*-thalassemia patient than DFO-only therapy [59, 60]. Reported side effects in this study include neutropenia, severe gastrointestinal disfunction, and arthropathy but did not warrant discontinuation of therapy. Therefore, combination therapies may be considered as an alternative to high-dose continuous conventional therapies for treatment of NDs due to a better QOL profile.

More recently, Feralex-G (FXG), a synthetic chelator, has been developed to chelate both cellular iron (Fe^3+^) and aluminum (Al^3+^) [61, 62]. It has also been reported that FXG contributes to scavenging and can repress reactive-oxygen-species- (ROS-) triggered gene expressions *in vitro* [63]. Although *in vivo* and *in vitro* investigations involving FXG will be required to completely evaluate its potential usefulness, the therapeutic potential of FXG in the treatment of NDs associated with iron-induced oxidative stress is promising.

M30 [5-(N-methyl-N-propargyaminomethyl)-8-hydroxyquinoline] and HLA20 (5-[4-propargylpiperazin-1-ylmethyl]-8-hydroxyquinoline) are multifunctional iron-chelating compounds that have been demonstrated to possess neuroprotective activities in mice ND models [64]. These novel drugs have the following three characteristic functions: iron chelation, free radical capture, and inhibition of iron-induced membrane lipid peroxidation. Their chemical structures contain the propargyl moiety of the monoamine oxidase-B (MAO-B) inhibitor drugs, rasagiline (Azilect), and the antioxidant iron-chelating moiety of VK28 (5-[4-(2-hydroxyethyl) piperazine-1-ylmethyl]-quinoline-8-ol) [65]. In ALS and PD models, M30 increased the rate of survivability and prevented the onset of neurological dysfunction [66, 67]. The neuroprotective effect of M30 treatment involves in hypoxia-inducible factor-1*α* (HIF-1*α*), a key stress-sensitive transcription factor that regulates transcription of the iron-regulatory, antioxidative, and glycolytic genes such as TfR, heme oxygenase-1, inducible nitric oxide synthase (iNOS), and glucose transporter-1 [64]. Mechanistically, the iron-chelating activity of M30 and HLA20 leads to inhibition of HIF prolyl-4-hydroxylases, an iron-dependent suppressor of HIF-1*α*. This inhibition subsequently activates HIF-1*α*-dependent cell survival pathways, such as mitogen-activated protein kinase (MAPK) and protein kinase C (PKC) pathways, accompanied by increases in growth-promoting factor levels, specifically those of brain-derived neurotrophic factor in the cortex and striatum, and the glial cell-derived neurotrophic factor in the hippocampus and spinal cord. Kupershmidt et al. also showed that in an ALS model, neurodegeneration induced by H_2_O_2_ and the oxynitrite generator 3-morpholino sydnonimine 1 (SIN-1) were significantly reduced by M30 and HLA 20 *in vitro* as demonstrated by reduced DNA fragmentation related to apoptotic cell death, when measured by enzyme-linked immunosorbent assay (ELISA). Moreover, these compounds selectively inhibit the activity of MAO-B, an enzyme that catalyzes the oxidative deamination of monoamines such as dopamine and noradrenalin, both of which are more specific substrates for MAOs than any other monoamines in the cerebral cortex [68]. Such monoamine metabolic reactions ultimately result in the production of H_2_O_2_, ammonia, and aldehydes, all of which have potentially neurotoxic effects at high levels. It follows that selectively inhibiting MAO can contribute to neuronal protection against redox substances. Thus, the novel iron chelator drugs, M30 and HLA20, potentially possess multiple pharmacological properties that culminate in antioxidative and neuronal protective effects that would help in restoring iron homeostasis in NDs.

## 5. Future Perspectives in ND Therapies

Although it appears that iron dysregulation is associated with NDs, and iron chelators are potentially effective drugs for the treatment of these diseases, the mechanisms of iron regulation under normal and abnormal conditions or its neuroprotective effects in the brain remain unclear. The common pathological features in NDs indicate that damage from oxidative stressors such as iron overload, H_2_O_2_, and endogenous neurotoxins primarily affect neurons in the brain. Recent clinical studies showed that iron chelators successfully capture iron in the brain and other tissues, thereby decreasing abnormal iron accumulation and inhibiting free radical production. However, conventional iron chelation therapies showed severe side effects that patients could not recover from, thereby indicating that safer and more effective drugs are required for the treatment of NDs. Combined iron chelation therapies are considered effective for the treatment of PD and AD, and they may also be useful for the treatment of other NDs such as ALS. Combination of an iron chelator with an MAO inhibitor provides neuroprotective effects in the brain, which could prevent apoptosis and activate cellular protective pathways against oxidative stresses. However, it is considered by some that despite the effectiveness of MAO inhibitors, products of monoamine metabolisms may contribute to neurotoxicity. It remains unclear whether the enzyme activity of MAOA and MAOB has cellular-, tissue-, and organism-specific differences [68]. Such heterogeneity in the activities of MAO indicates that further knowledge about redox regulatory mechanisms in the brain is required for its clinical application in the treatment of ND.

The brain comprises not only neurons but also glial cells, which maintain internal brain homeostasis by supporting neuron structures, regulating the clearance of neurotransmitters, and releasing gliotransmitters. There have been several reports on neuroprotection against oxidative stressors by glial cells [69, 70]. In these reports, astrocytes were shown to preserve neurons from ROS- and nitrogen species-induced neuronal damages. Some reports indicate that microglia also prevent NO-induced neuronal apoptosis by secreting heat-labile and -stable neuroprotective factors *in vitro* [70]. However, other reports have shown that microglia cause neuronal cell death by secreting neurotoxic substances including ROS [71, 72]. At first glance, these reports appear conflicting as they state that microglia mediate both neuroprotection and neuronal cell death. However, both studies make sense when one takes into account the possibility that microglia potentially have multiple roles, including their functions as macrophages that regulate immune and inflammatory responses. Interestingly, their immunological activities are regulated by iron fluxes mediated by hepcidin and FPN [49]. Thus, there is a high possibility that drugs for NDs affect not only neurons but also glial cells.

Currently, several *in vitro* studies suggest that the brain has potential antioxidative abilities that can protect cells against oxidative damage and maintain brain homeostasis. However, knowledge about endogenous antioxidative mechanisms in the brain remains insufficient to improve noninherited heterogeneous NDs that result from complicated anti-/pro-oxidative molecular system dysfunctions. Thus, clinical studies on the biological significance of drug interactions and on experimental evidence for iron-mediated oxidative stresses in the brain are necessary. Further research advances will aid in the development of novel, effective, molecule/cell-targeted, and safer drugs for NDs.
